# Parity as a Risk Factor for Differentiated Thyroid Carcinoma: A Population-based Study

**DOI:** 10.1210/clinem/dgaf642

**Published:** 2025-11-24

**Authors:** Rotem Dan, Nave Paran, Nuphar Vinegrad, Merav Fraenkel, Uri Yoel

**Affiliations:** The Goldman Medical School at the Faculty of Health Sciences, Ben-Gurion University of the Negev, 84105 Beer Sheva, Israel; Clinical Research Center, Soroka University Medical Center, 84101 Beer Sheva, Israel; Faculty of Health Sciences, Ben-Gurion University of the Negev, 84105 Beer Sheva, Israel; Endocrinology Unit, Soroka University Medical Center, 84101 Beer Sheva, Israel; Faculty of Health Sciences, Ben-Gurion University of the Negev, 84105 Beer Sheva, Israel; Endocrinology Unit, Soroka University Medical Center, 84101 Beer Sheva, Israel; Faculty of Health Sciences, Ben-Gurion University of the Negev, 84105 Beer Sheva, Israel; Endocrinology Unit, Soroka University Medical Center, 84101 Beer Sheva, Israel

**Keywords:** differentiated thyroid cancer, papillary thyroid carcinoma, parity, multiparity

## Abstract

**Context:**

Female sex is a risk factor for differentiated thyroid carcinoma (DTC), potentially due to reproductive influences. However, data on the association between parity and DTC risk remain inconsistent.

**Objective:**

To assess the association between parity and DTC risk in a high-multiparity population.

**Design:**

Population-based case-control study (1982-2022).

**Setting:**

A single tertiary medical center.

**Patients or Other Participants:**

The study included 300 female patients with DTC and 900 controls, matched by birth year and ethnicity. The primary exposure was the number of deliveries before DTC diagnosis.

**Main Outcome Measure(s):**

Association between parity and DTC risk evaluated by logistic regression, adjusted for socioeconomic status, TSH levels, oral contraceptive use, and autoimmune thyroid diseases.

**Results:**

The median age at DTC diagnosis was 39 years; 60% of participants were Jewish and 40% were Arab. Baseline characteristics were comparable, except for higher rates of autoimmune thyroid diseases in cases: Hashimoto thyroiditis (9.7% vs 1.8%, *P* < .001) and Graves’ disease (6.3% vs 2.7%, *P* = .005). Parity was associated with increased DTC risk starting at 4 deliveries (OR = 1.70, 95% CI: 1.051-2.741, *P* = .030), with the highest risk at 6 or more (odds ratio = 1.89; 95% CI, 1.052-3.393; *P* = .033). This association was largely driven by Arab women, who had significantly higher grand multiparity rates (62.6% vs 13.4%; median 5 vs 3 deliveries; *P* < .001).

**Conclusion:**

High parity, primarily among Arab women, was associated with increased DTC risk, with significance observed at 4 or more deliveries.

Differentiated thyroid cancer (DTC) refers to thyroid neoplasms derived from follicular cells, including papillary, follicular, and oncocytic thyroid cancers (TC). Papillary TC (PTC), the most common histologic subtype, accounts for about 90% of new cases, and has the best prognosis, with a 10-year overall survival rate >90% (1, 2). Established risk factors for DTC include female gender, a history of exposure to ionizing radiation, mainly in childhood; a family history of DTC; and an elevated TSH level (3, 4). Obesity has recently been suggested as a risk factor, mainly for PTC (1, 5).

As DTC is 3 times more common in females (6), reproductive factors have been suggested to have an important role in its pathogenesis (1, 2). Epidemiological investigations of the association between reproduction parameters and DTC have yielded inconsistent results. A nationwide South Korean study reported associations of pregnancy, parity, and the number of reproductive years with DTC, after adjustment for age, body mass index (BMI), and smoking status (7). Likewise, a meta-analysis that included 8860 women found a statistically significant association between parity and the risk of TC (relative risk for parous vs nulliparous: 1.09; 95% CI, 1.03-1.15; *I*²=33.4%) (8). In contrast, a case control study that included 430 cases of DTC and 505 controls found that parity (yes or no) was not associated with the risk of TC (9). Importantly, a cohort study and meta-analysis by LeClair et al challenged the longstanding paradigm that TC is inherently more common among women (10). The authors demonstrated that, in autopsy studies, small subclinical PTCs were detected at similar rates in both sexes. They suggested that the reported female predominance in clinically diagnosed PTC may reflect overdetection in women, rather than a true gender-specific predisposition. This perspective questions the role of female-specific factors, such as reproductive history, in the etiology of DTC, and underscores ongoing controversy in the field.

Soroka University Medical Center provides health services for a population of more than 1 million. Of the 17 000 annual live births, the proportion delivered by grand multiparous women is relatively high. In this unique population, we aimed to investigate the contribution of reproductive factors, specifically parity, on DTC risk. We hypothesized that parity is positively linked to an increased risk of DTC.

## Materials and Methods

### Study Population

**Figure 1.**
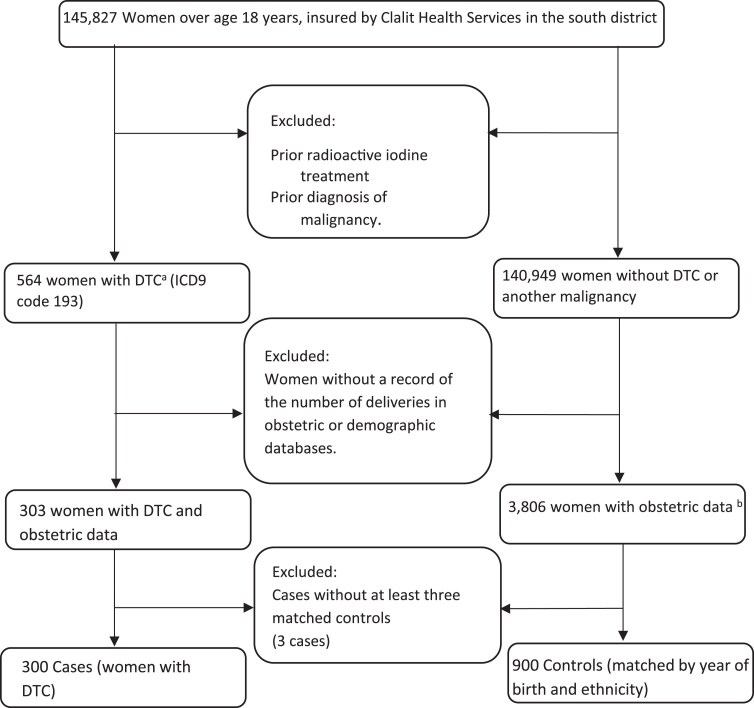
^a^Women diagnosed with DTC between January 1982 and December 2022. ^b^Women with obstetric data, matched by year of birth and ethnicity to one of the cases.

In this population-based case-control study, we screened the electronical medical records of females aged 18 years and older, insured by Clalit Health Services in the southern district of Israel. The cases group comprised those who were diagnosed with DTC (International Classification of Diseases, 9th revision [ICD9] code 193) between January 1982 and December 2022. The control group comprised 3 females from the same database, per each “case,” matched by year of birth and ethnicity (Jewish or Arab). For both groups, women were excluded if they had a prior malignancy (other than DTC) or prior radioactive iodine treatment for nonmalignant thyroid disease (Fig. 1). The study was approved in advance by the institutional ethical committee (approval number SOR-0003-18). In accordance with national guidelines, the requirement for informed consent was waived due to the retrospective study design.

### Definition of the Event Date

After all the patients diagnosed with DTC were identified, we performed another screen for “thyroid” and “thyroidectomy” in each patient's records. For those with a record of thyroidectomy before the first ICD9 code 193 record, we considered the date of the pathology report of DTC as the event date. For all the other patients in the case group, the event date was the earliest date of the DTC diagnosis in the electronic medical record. For control patients, the event date was set to match the event date of their corresponding case, ensuring that exposure assessment for controls was aligned with the same point in time as for the cases.

### Definition of Exposure

The primary exposure was the number of deliveries (live births), as recorded in the obstetric database of our center. Nulliparity (zero deliveries) was defined by the indication of “no children” in the demographic database and an absence of birth records in the obstetric database. We excluded women without documented labor in the obstetric database and missing a confirmation in the demographic database (Fig. 1). The number of deliveries was retrieved from the obstetric database, from the last birth record before the event date.

### Data Sources

Demographic, clinical, biochemical, and relevant drug purchase data of cases and controls were retrieved from the database of Clalit Health Services’ southern district. Obstetrical data were retrieved from the obstetric database of our center. In this study, we retrieved and analyzed from these databases only data that were recorded before the event date.

The data that were retrieved from the database of the southern district of Clalit Health Services included, though were not limited to, age (years), ethnicity (Jewish/Arabs), the number of children, smoking status (yes/no), BMI (the last nonpregnant value before the event date), a past diagnosis of Graves’ disease or Hashimoto thyroiditis (based on ICD9 codes), and TSH (mIU/L) laboratory results. TSH levels were evaluated 2 to 5 years before the event date. This strategy was chosen to avoid recording an extremely elevated TSH level, which can appear during levothyroxine withdrawal before radioactive iodine treatment, among patients whose DTC diagnosis was coded later than at the time of the clinical diagnosis. Oral contraceptive purchasing data (ATC G03A) (any/no) were also retrieved from the Clalit Health Services database. Socioeconomic status was based on the Israeli Central Bureau of Statistics socioeconomic score, ranging between 1 and 10, with 1 being the lowest socioeconomic score and 10 the highest (11).

Obstetric parameters including maternal age at the first delivery (years), a diagnosis of gestational diabetes mellitus in all documented pregnancies (yes/no), newborn weight in all deliveries (g), and multiple pregnancies (yes/no) were retrieved from the obstetrics database of our center. Assisted reproductive fertility (yes/no) was based on records of specific procedures from the fertility center database. The selected procedures were those that potentially comprised hormonal treatments and are detailed in Table S1 (12).

### Statistical Analyses

Baseline demographic, clinical, biochemical, and obstetrics characteristics were summarized using descriptive statistics. Continuous variables are presented as mean ± SD; and as median, 25th percentile, and 75th percentile. Categorical variables are presented as counts and percentages. We used Pearson's Chi-squared test to compare the categorical variables between the cases and control groups, and the Mann-Whitney test and *t*-test to compare the continuous variables. We used the Mann-Whitney test (Wilcoxon rank-sum test with continuity correction) to compare the number of deliveries and the number of pregnancies.

We evaluated the independent association between DTC and the number of deliveries using multivariable conditional logistic regression analysis. Exposure was defined by 2 indicators: 1 for nulliparity (yes/no) and the other for the number of deliveries. To determine the threshold at which parity becomes significantly associated with DTC risk, we conducted a structured series of multivariable logistic regressions using incrementally increasing parity categories as reference points (eg, 1 delivery vs 2 or more, 2 vs 3 or more, and so on, up to 5 vs 6 or more deliveries). This approach was prespecified and designed to assess a potential dose-response relationship, rather than to test multiple independent hypotheses. Therefore, correction for multiple testing was not deemed necessary and done as a part of a sensitivity analysis. We first analyzed the association between DTC and parity for all the cases and controls. Then, we conducted stratified analyses for the 2 main ethnic groups in our population, Jews and Arabs, who differ significantly in their reproductive patterns. Specifically, Arab women had a higher median number of deliveries and markedly greater rates of multiparity and grand multiparity compared to Jewish women, as shown in Table S2 (12). These differences in parity distribution could potentially modify the strength or detectability of the association between parity and DTC risk in each subgroup. In the multivariable analysis, we adjusted for the following potential confounders: socioeconomic status, mean TSH levels, prior diagnoses of Hashimoto or Graves' disease, year of diagnosis, and the use of oral contraceptives. A 2-sided *P* value of .05 or lower was considered statistically significant. Statistical analysis was performed by “R” statistical software, version 4.2.0 for windows, and graph production by the “GraphPad” software version 10.2.

## Results

### Baseline Characteristics

Our study included 300 women diagnosed with DTC, designated as cases, and a control group of 900 women matched by year of birth and ethnicity (Fig. 1). The median age at the diagnosis of DTC among the cases was 39 years (range, 21-65). In general, baseline characteristics were comparable between the case and control groups (Table 1). The median values of socioeconomic status index were 3 and 4, respectively, of 10 (*P* = .415). The mean BMI (the last nonpregnant measurement before the event date) was about 27 in both groups (*P* = .802). The mean TSH level at 2 to 5 years before the diagnosis was 2.39 mIU/L for the case group and 2.21 mIU/L for the control group (*P* = .427). Autoimmune thyroid diseases were more prevalent in the cases group: Hashimoto thyroiditis, 9.7% vs 1.8% (*P* < .001); and Graves’ disease, 6.3% vs 2.7% (*P* = .005). Table 2 presents the obstetrics characteristics. For both groups, the median age at the first delivery was 26 years, and the median number of deliveries was 3 (range, 0-15). Neither clinical nor statistical differences were found between cases and control groups in the rates of multiple pregnancies, gestational diabetes mellitus, the need for assisted reproductive fertility, and oral contraceptive use. The mean newborn weight of all the deliveries was slightly higher for the cases than the control group (3216 ± 480 g vs 3132 ± 478 g, *P* = .012).

**Table 1.** Baseline characteristics of women with differentiated thyroid cancer (cases) and matched controls

	Case N = 300	Controls N = 900	*P^a^*
Age, years			.989
Mean ± SD (n)	40 ± 10 (300)	40 ± 9 (900)	
Median	39	39	
Min; Max	21, 65	21, 65	
Ethnicity, n/N (%)			1.000
Arabic	120/300 (40.0%)	360/900 (40.0%)	
Jewish	180/300 (60.0%)	540/900 (60.0%)	
Socioeconomic status			.415
Mean ± SD (n)	4 ± 3 (298)	3 ± 3 (897)	
Median	4	3	
Min; Max	0, 10	0, 10	
Smoking status in the past or present			.567
n/N (%)	40/211 (19.0%)	189/899 (21.0%)	
Diabetes mellitus type 2			.232
n/N (%)	28/300 (9.3%)	63/1200 (7.0%)	
BMI			.802
Mean ± SD (n)	27.3 ± 6.2 (184)	27.5 ± 6.2 (584)	
Median	27.0	26.7	
Min; Max	17.6, 59.5	16.2, 56.2	
TSH levels, 2-5 years before event date, mIU/L			.427
Mean ± SD (n)	2.39 ± 2.90 (186)	2.21 ± 1.59 (730)	
Median	1.79	1.88	
Min; Max	0.17, 27.42	(0.01, 20.72)	
Hashimoto thyroiditis, n/N (%)	29/300 (9.7%)	16/900 (1.8%)	<.001
Graves' disease, n/N (%)	19/300 (6.3%)	24/900 (2.7%)	.005

Abbreviations: BMI, body mass index; Max, maximum; Min, minimum.

^*a*^Pearson's chi-squared test; Welch 2-sample *t*-test.

**Table 2.** Obstetrics characteristics of women with differentiated thyroid cancer (cases) and matched controls

	Cases N = 300	Controls N = 900	*P^a^*
Age at first delivery*^b^*, years			.690
Mean ± SD (n)	27.1 ± 5.9 (273)	27.0 ± 5.5 (850)	
Median	26.3	26.2	
Q1;Q3	22.3, 31.3	22.6, 30.7	
Number of pregnancies*^b^*			.120
Mean ± SD (n)	5 ± 3 (273)	5 ± 3 (850)	
Median	4	4	
Q1;Q3	3, 7	3, 6	
Number of deliveries*^b^*			.337
Mean ± SD (n)	4 ± 3 (300)	4 ± 3 (900)	
Median	3	3	
Q1;Q3	2, 6	2, 5	
Multiple pregnancies*^b^*, n/N (%)	12/273 (4.4%)	50/850 (5.9%)	.433
Newborn weight in all the deliveries*^b^*, g			.012
Mean ± SD (n)	3216 ± 480 (273)	3132 ± 478 (850)	
Median	3226	3162	
Q1;Q3	2,960, 3535	2,866, 3446	
Diagnosis of gestational diabetes mellitus in any documented pregnancy*^b^*, n/N (%)	34/273 (12.5%)	104/850 (12.2%)	.999
Assisted reproductive fertility procedure cycle*^c^*, n/N (%)	13/300 (4.3%)	28/900 (3.1%)	.409
Use of oral contraceptives, n/N (%)	121/300 (40.3%)	422/900 (46.9%)	.056

Abbreviation: Q, quartile.

^*a*^Pearson's chi-squared test; Wilcoxon rank-sum test with continuity correction.

^*b*^N includes only patients with an obstetrics record. Nulliparous were excluded from these analyses.

^c^The procedures are detailed in Table S1.

The cohort comprised 2 ethnic groups (60% Jewish, 40% Arab). Baseline characteristics by ethnicity are summarized in Table S3 (12). Compared with Jewish women, Arab women were younger at diagnosis (median, 37 vs 41 years; *P* < .001), had lower socioeconomic status (median SES index, 1 vs 5-6; *P* < .001), higher diabetes prevalence (eg, among controls: 11% vs 4.6%; *P* = .001), and higher BMI. In contrast, Jewish women were more likely to smoke and use oral contraceptives and tended to begin childbearing later (mean age at first delivery ∼29 vs ∼25 years; *P* < .001).

### Parity and the Risk of DTC

**Figure 2.**
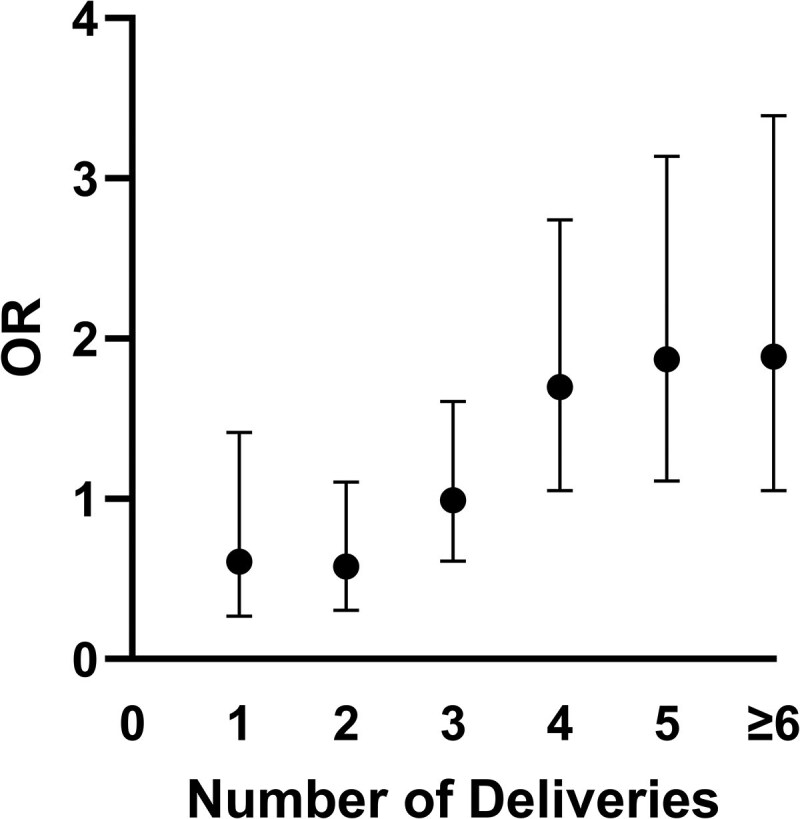
The association between the number of deliveries and DTC risk is presented as odds ratios (OR). The risk for DTC increases with the number of deliveries, becomes statistically significant for 4 deliveries and above.

In our primary analysis that focused on parity, the median number of deliveries between the 2 groups did not differ significantly. Compared with the controls, the cases group was characterized by a higher rate of nulliparous (9.0% vs 5.6%) and of women with more than 6 deliveries (22.0% vs 16.0%), as shown in Table S4 (12). In the multivariable regression that adjusted for potential confounders, including socioeconomic status, TSH levels, oral contraceptive usage, rates of autoimmune thyroid diseases, and year of diagnosis, 1 or more deliveries compared to nulliparity did not increase the risk of DTC. The same was true when 1 to 2 deliveries served as a reference and was compared with a higher number of deliveries (Table 3). On the other hand, the association between parity and DTC risk was statistically significant, starting with 4 or more deliveries vs a reference of 1 to 3 deliveries (odds ratio [OR] = 1.70; 95% CI, 1.051-2.741; *P* = .030). The highest OR was for women with 6 or more deliveries compared to women with 1 to 5 deliveries as a reference (OR = 1.89; 95% CI, 1.052-3.393; *P* = .033) (Fig. 2, Tables 3 and 4).

**Table 3.** Multivariable analyses for associations between various numbers of deliveries and the risk of differentiated thyroid cancer

	OR	95% CI	*P*
Parity (≥1 deliveries) vs Nulliparity	0.61	0.267-1.415	.252
Nulliparity	0.86	0.369-2.760	.985
Reference (1 delivery)	1.00		
High parity (≥2 deliveries)	0.58	0.305-1.104	.097
Nulliparity	1.62	0.672-3.911	.282
Reference (1-2 deliveries)	1.00		
High parity (≥3 deliveries)	0.99	0.613-1.608	.978
Nulliparity	1.98	0.836-4.702	.120
Reference (1-3 deliveries)	1.00		
High parity (≥4 deliveries)	1.70	1.051-2.741	.030
Nulliparity	1.90	0.810-4.463	.140
Reference (1-4 deliveries)	1.00		
High parity (≥5 deliveries)	1.87	1.113-3.138	.018
Nulliparity	1.72	0.741-4.004	.206
Reference (1-5 deliveries)	1.00		
High parity (≥6 deliveries)	1.89	1.052-3.393	.033

The analyses were adjusted for socioeconomic status, the use of oral contraceptives, TSH level, year of diagnoses, and a diagnosis of Graves’ disease of Hashimoto thyroiditis.

Abbreviations: C.I., confidence interval; OR, odds ratio.

**Table 4.** Multivariable analyses for associations of 6 or more compared to 1-5 deliveries, with the risk of differentiated thyroid cancer

Adjustment	OR	95% CI	*P*
a. No adjustment			
Nulliparity	2.04	1.181-3.539	.011
Reference (1-5 deliveries)	1.00		
High parity (≥6 deliveries)	1.73	1.161-2.585	.007
b. Adjusted for SES*^a^*			
Nulliparity	1.94	1.113-3.390	.019
Reference (1-5 deliveries)	1.00		
High parity (≥6 deliveries)	1.76	1.161-2.634	.006
c. Adjusted for OC*^b^* and SES			
Nulliparity	1.85	1.057-3.245	.031
Reference (1-5 deliveries)	1.00		
High parity (≥6 deliveries)	1.76	1.174-2.631	.006
d. Adjusted for “c” and TSH*^c^*			
Nulliparity	1.63	0.756-3.230	.213
Reference (1-5 deliveries)	1.00		
High parity (≥6 deliveries)	1.90	1.118-3.509	.018
e. Adjusted for “d” and a diagnosis of HT/GD			
Nulliparity	1.61	0.721-3.587	.246
Reference (1-5 deliveries)	1.00		
High parity (≥6 deliveries)	2.18	1.233-3.853	.007
f. Adjusted for “e” and year of diagnosis			
Nulliparity	1.722	0.741-4.004	.206
Reference (1-5 deliveries)	1.00		
High parity (≥6 deliveries)	1.890	1.052-3.393	.033

Abbreviations: GD, Graves’ disease; HT, Hashimoto thyroiditis; OC, oral contraceptives; OR, odds ratio; SES, socioeconomic status.

^*a*^According to the Israeli Central Bureau of Statistics socioeconomic score.

^*b*^Any purchase of OC.

^*c*^Average of all TSH results 5 to 2 years before DTC diagnosis.

### Subgroup and Stratified Analyses

The Arab population was characterized by higher parity than the Jewish population, as shown in Tables S5 and S6 (12). Among the Arab women, 40.0% had more than 6 deliveries compared to only 2.8% among the Jewish women. Conversely, Jewish women demonstrated higher rates of nulliparity (7.1% vs 5.4%). The median number of deliveries was 3 for Jewish and 5 for Arab women (*P* < .001), as shown in Table S2 (12). In ethnicity-specific multivariable logistic regression (Table 5), we used the same strategy as in the full cohort. To reflect the different parity distributions, the reference category was 1 to 4 deliveries for Jewish women and 1 to 5 deliveries for Arab women. As shown in Table S2 (12), ≥6 deliveries occurred in 6.7% of Jewish women (48/720), whereas ≥5 deliveries occurred in 62.6% of Arab women (300/480). We therefore defined high parity as ≥5 for Jewish women and ≥6 for Arab women to capture the upper tail in each group while retaining adequate numbers for stable estimates.

**Table 5.** Multivariable analysis of the association between the number of deliveries and the risk of differentiated thyroid cancer, stratified by ethnicity

Jewish population			
Adjustment	OR	95% CI	*P*
a. No adjustment			
Nulliparity	2.30	1.155-4.586	.018
Reference (1-4 deliveries)	1.00		
High parity (≥5 deliveries)	1.12	0.668-1.887	.665
b. Adjusted for SES*^a^*, OC*^b^*, TSH*^c^*, HT/GD, YOD*^d^*			
Nulliparity	1.32	0.504-3.449	.573
Reference (1-4 deliveries)	1.00		
High parity (≥5 deliveries)	1.36	0.804-2.297	.252
**Arab population**			
**Adjustment**	**OR**	**95% CI**	***P* value**
a. No adjustment			
Nulliparity	2.04	0.737-4.835	.185
Reference	1.00		
High parity (≥6 deliveries)	2.57	1.484-4.464	.0008
b. Adjusted for SES*^a^*, OC*^b^*, TSH*^c^*, HT/GD, YOD*^d^*			
Nulliparity	2.58	0.645-10.262	.178
Reference	1.00		
High parity (≥6 deliveries)	4.40	1.686-11.482	.002

High parity was defined as 5 deliveries or more among Jewish women, and as 6 deliveries or more among Arab women.

Abbreviations: GD, Graves’ disease; HT, Hashimoto thyroiditis; OC, oral contraceptives; OR, odds ratio; SES, socioeconomic score; YOD, year of diagnosis.

^*a*^According to the Israeli Central Bureau of Statistics socioeconomic score.

^*b*^Any purchase of OC.

^*c*^Average of all the TSH results 5 to 2 years before the diagnosis of differentiated thyroid cancer.

^*d*^Year of diagnosis.

Following adjustment for potential confounding factors, no significant association was observed between any number of deliveries and an increased DTC risk among Jewish women. In contrast, among Arab women, DTC risk was associated with 6 deliveries or more (OR = 4.40; 95% CI, 1.686-11.482; *P* = .002), compared with a reference of a lower number of deliveries (Table 5, Tables S7 and S8) (12).

As part of sensitivity analysis we applied Benjamini-Hochberg false discovery rate correction to the ordered parity contrasts. In the full cohort, the 3 contrasts that were significant before correction (≥4 vs 1-3; ≥5 vs 1-4; ≥6 vs 1-5) retained the same direction and were near significance after correction (q(BH) = 0.066 for each; Table S9) (12). Among Arab women, the association for ≥6 vs 1 to 5 remained statistically significant after correction (q(BH) = 0.012; Table S10) (12).

## Discussion

This population-based case-control study demonstrated a positive association between high parity and the risk of DTC, with the increased risk becoming statistically and clinically significant at 4 or more deliveries, and the highest OR observed at 6 or more deliveries (OR = 1.89; 95% CI, 1.05-3.39). In contrast, similar associations were not found for either the comparison between nulliparous women and those who experienced 1 delivery or more, or for 1 or 2 deliveries as a reference compared with a higher number of deliveries. Notably, this association was primarily driven by Arab women, among whom grand multiparity was far more prevalent than among Jewish women (62.6% vs 13.4%; median 5 vs 3 deliveries; *P* < .001). Indeed, subgroup analysis revealed a strong association of parity with DTC risk among Arab women, with 6 or more deliveries (OR = 4.40; 95% CI, 1.69-11.48).

Our findings align with a large meta-analysis of 23 studies including 8860 women, which demonstrated a significant association between parity and the risk of DTC (relative risk = 1.09; 95% CI, 1.03-1.15; *I*^2^ = 33.4%). This positive association persisted in almost all the strata of the subgroup analysis, although statistical significance was not detected in all of them (8). In another study, based on the Korea National Health and Nutrition Examination Survey and including 21 534 females with 210 DTC cases, a positive association between parity and the risk of DTC was found (OR = 7.60; 95% CI, 3.02-19.13; *P* < .01). The authors also found that DTC incidence was increased as the number of pregnancies increased (7). That study was based on a self-reported questionnaire, which may have affected data reliability.

Our hypothesis that parity increases the risk of DTC was grounded in biological mechanisms. Several studies have suggested a link between human chorionic gonadotropin, which is produced in large quantities by the placenta during pregnancy, and increased DTC risk. This may be related to human chorionic gonadotropin's ability to activate the TSH receptor, thereby increasing thyroid hormone synthesis and secretion, and inducing hypertrophic and hyperplastic effects on the thyroid gland (13, 14). Another possible mechanism involves high cumulative estrogen exposure during and after pregnancy. Estrogen acts as a potent growth factor for DTC cells. In vitro studies have indicated that abnormal estrogen metabolism may enhance metastatic potential in PTC cell lines (15, 16). Finally, the increase in human placental lactogen during pregnancy stimulates the production of IGF-1, which may also link reproductive factors with DTC. IGF-1 has pro-carcinogenic effects, and its receptors are overexpressed in DTC cells (17).

In contrast to this, the findings of some studies do not support an association between parity and DTC risk (9, 18, 19). Interestingly, studies that found no link between parity and DTC risk were conducted in populations with parity patterns similar to those of the Jewish women in our study, for whom a statistically significant association was also not demonstrated. In a large, recently published, pooled prospective analysis of cohorts from North America, Europe, Australia, and Asia, comprising 1 252 907 women of whom 2124 were diagnosed with DTC, O’Grady et al (18) reported no association between parity and DTC. The mean parity in O’Grady's study (1.5-3.1) closely mirrored the mean parity of 3 in the Jewish population in our study. In contrast, among Arab women in our study, who were characterized by a higher rate of multiparity, a clinically and statistically significant association between parity and DTC risk was observed. We suggest that the association between parity and DTC risk exists but is more pronounced with a higher number of deliveries, which is often not seen in developed/high-income countries (20).

We report similar DTC risk among nulliparous women and those who had given birth. Notably, the cases group comprised a higher proportion of nulliparous women than the control group (27/300, 9.0% vs 50/900, 5.6%), which exceeded rates reported in other studies (7, 19). However, those studies relied on self-reported data regarding diagnoses and childbirth dates. One possible explanation for the higher rate of nulliparous women in the cases group is the increased prevalence of autoimmune thyroid diseases, which may delay childbearing age. Additionally, as part of the diagnostic process for thyroid disorders, women often undergo thyroid ultrasounds, potentially leading to higher rates of DTC diagnosis (21, 22).

Our study has several limitations. First, the size of our population was insufficient to facilitate a thorough subgroup analysis. Second, discrepancies may exist between the actual date of DTC diagnosis and its record in the electronic medical file, particularly for women diagnosed before 2004, the year that medical records became universally computerized. This gap could create a situation in which a delivery was recorded before the DTC diagnosis, even though the diagnosis occurred earlier. However, as we employed advanced computerized tools, we presume that this potential bias, if it exists, is minimal. Finally, the number of deliveries might be inaccurate for women who gave birth in other hospitals, potentially leading to an underestimation of their deliveries. However, this potential bias equally affects the cases and control groups and thus seems unlikely to attenuate the results.

The main strength of our study is our ability to investigate a population with a substantial proportion of grand multiparous women. Furthermore, our access to the database of Israel's largest health maintenance organization enabled establish a customized cohort database that encompassed all the necessary data from outpatient clinics and our center. This facilitated seamless cross-linking between data sources, ensuring the availability of comprehensive patient information. Our screening process was uniformly implemented across our customized database, thereby ensuring consistent screening procedures for both the cases and control groups.

In summary, our study findings contribute further evidence for the association between parity and DTC risk. Like previous studies, we observed a nonlinear positive relation between parity and DTC risk, underscoring the need for additional research to elucidate its underlying mechanisms.

## Data Availability

Data are available on request and according to the national policy of data sharing requiring authorization by the ethics committee.

## Abbreviations

BMI: body mass index
DTC: differentiated thyroid cancer
ICD9: International Classification of Diseases, 9th revision
OR: odds ratio
PTC: papillary thyroid cancer
TC: thyroid cancer
